# Prokaryotic Caspase Homologs: Phylogenetic Patterns and Functional Characteristics Reveal Considerable Diversity

**DOI:** 10.1371/journal.pone.0049888

**Published:** 2012-11-19

**Authors:** Johannes Asplund-Samuelsson, Birgitta Bergman, John Larsson

**Affiliations:** Department of Botany, Stockholm University, Stockholm, Sweden; Loyola University Medical Center, United States of America

## Abstract

Caspases accomplish initiation and execution of apoptosis, a programmed cell death process specific to metazoans. The existence of prokaryotic caspase homologs, termed metacaspases, has been known for slightly more than a decade. Despite their potential connection to the evolution of programmed cell death in eukaryotes, the phylogenetic distribution and functions of these prokaryotic metacaspase sequences are largely uncharted, while a few experiments imply involvement in programmed cell death. Aiming at providing a more detailed picture of prokaryotic caspase homologs, we applied a computational approach based on Hidden Markov Model search profiles to identify and functionally characterize putative metacaspases in bacterial and archaeal genomes. Out of the total of 1463 analyzed genomes, merely 267 (18%) were identified to contain putative metacaspases, but their taxonomic distribution included most prokaryotic phyla and a few archaea (Euryarchaeota). Metacaspases were particularly abundant in Alphaproteobacteria, Deltaproteobacteria and Cyanobacteria, which harbor many morphologically and developmentally complex organisms, and a distinct correlation was found between abundance and phenotypic complexity in Cyanobacteria. Notably, *Bacillus subtilis* and *Escherichia coli*, known to undergo genetically regulated autolysis, lacked metacaspases. Pfam domain architecture analysis combined with operon identification revealed rich and varied configurations among the metacaspase sequences. These imply roles in programmed cell death, but also e.g. in signaling, various enzymatic activities and protein modification. Together our data show a wide and scattered distribution of caspase homologs in prokaryotes with structurally and functionally diverse sub-groups, and with a potentially intriguing evolutionary role. These features will help delineate future characterizations of death pathways in prokaryotes.

## Introduction

### Programmed Cell Death in Prokaryotes

Programmed cell death (PCD) is involved in numerous important stages of metazoan life, including tissue homeostasis, embryo development, defense against viral pathogens and elimination of cancer cells [1], [2]. In order to reach and maintain the structure of a complex, multicellular organism, it is necessary to control the behavior of individual cells, and a means to do this is via a tightly regulated and controlled self-destruction. Likewise, organisms within the plant kingdom utilize PCD in various contexts (see [3]), although their physical fixation gives rise to alternative pathways, such as the hypersensitive response to pathogens [4], during wood (xylem) formation [5] and reproductive development [6]. Since the turn of the century, it has become increasingly clear that morphologically less complex, and even unicellular organisms, including prokaryotes, may initiate specific cell death programs [7]–[13].

Prokaryotes, largely single-celled organisms, may reap evolutionary benefits from behaviors that directly favor the survival of clones rather than the individual cell performing them, i.e. a form of altruism. For instance, sacrifice of a subset of the cells through genetically controlled death would in certain situations allow the remaining to re-initiate growth and perpetuate the genotype, rather than going extinct. This type of behavior has been observed in bacterial strains in response to stress [14], [15], in developmental processes [8], [16] and in defense against phage infection [17] or antibiotics [18]. It is also recognized as an important factor in the development of biofilms [12], [19].

*Bacillus subtilis* deploys PCD in conjunction with spore formation, in the form of autolysis of the mother cell leading to release of the endospore [8]. The *Streptococcus pneumoniae* VncS protein is part of a pathway that controls antibiotic-induced autolysis, in order to eliminate damaged cells, a response that may be interpreted as PCD [8]. *Escherichia coli* may undergo PCD under control of the toxin-antitoxin system *mazEF* [18]. Such potentially destructive genetic modules are widely distributed among prokaryotes and come in many variants, e.g. *relBE* and *hipBA*, in addition to *mazEF,* in *E. coli* [20]. The toxin MazF, a stable RNAse, is antagonized by a labile antitoxin, MazE. PCD ensues if expression of the *mazEF* module seizes, since the toxin will remain active beyond that of the antitoxin, and is a response to stress and a defense against phage P1 [18]. PCD also plays a role in the development of fruiting bodies and myxospores in the social predatory bacterium *Myxococcus xanthus* DK 1622. During sporulation, 80% of the population is killed in a tightly regulated PCD process, via inactivation of a protein kinase cascade and an activation of the MazF-mx ribonuclease [16], [21]. The *M. xanthus* autolysis process, which is likely to depend on a set of autolytic enzymes, complementing the effects of the MazF-mx ribonuclease, has not been characterized [16]. Curiously, PCD in the Gammaproteobacterium *Xanthomonas campestris* [15], [22] and the Cyanobacterium *Trichodesmium erythraeum* IMS101 [14], is accompanied by the expression of “caspases”, ordinarily known as the central executioners in metazoan PCD pathways.

PCD in *X. campestris* pv. *glycines* occurs in the post-exponential growth phase and is associated with features characteristic of metazoan apoptosis [15], [22], for example annexin V binding to the plasma membrane, expression of caspases, and cleavage of a caspase-specific substrate. Gautam and Sharma [22] showed that caspase-deficient mutants were unable to undergo PCD, providing further evidence in favor of the importance of prokaryotic caspase homologs. More recently, Wadhawan and colleagues [15] identified metabolic and oxidative stresses as triggering factors for caspase expression, caspase activation and PCD in *X. campestris* pv. *glycines*.

The marine Cyanobacterium *T. erythraeum* IMS101 was shown to undergo PCD in response to conditions mimicking natural surface “bloom” conditions [14]. This was accompanied by the expression and activation of caspase proteins and increased nuclease activity, while plasma membrane integrity was retained. The unicellular Cyanobacterium *Microcystis aeruginosa* also exhibits caspase activity in response to induced oxidative stress [23].

### Metacaspases

Caspases, i.e. cysteine-dependent aspartate-directed proteases, are the central executioners of metazoan PCD [2], [24], [25]. An active site containing a critical cysteine-histidine catalytic dyad provides the proteolytic activity, and is considered the defining feature of caspases [26]. Aravind and co-workers were the first to detect caspase homologs in bacteria [27] and Uren and co-workers [28] were able to identify homologs in single-celled eukaryotes, in plants and in some bacteria. These homologs were divided into paracaspases and metacaspases, based on domain structure and sequence similarity. For simplicity, we here refer to all non-metazoan caspase homologs as metacaspases, regardless of their classification.

Metacaspases have been implicated in PCD in fungi [29], in plants [29], [30] and, as mentioned above, in some bacteria [14], [15], [22]. However, the similarity of metacaspases to caspases has been disputed, primarily due to the observation that metacaspases possess a substrate specificity (cleavage at arginine or lysine) different from that of caspases (cleavage at aspartic acid), and may not be responsible for the caspase activity detected in non-animal organisms undergoing PCD [25], [31], [32].

Over the years, metacaspases have been observed in multiple bacterial genomes, e.g. *Streptomyces*, *Pseudomonas*, *Mesorhizobium*, *Myxococcus*, *Xyllella*, *Anabaena*, *Synechocystis* and *Trichodesmium* [28], [33], [34]. In general, developmentally complex prokaryotes, as found in Cyanobacteria, Alphaproteobacteria and Actinobacteria, appear to have a large number of apoptotic protein homologs [33].

As of today, Pfam, a manually curated database of protein families (http://pfam.sanger.ac.uk/), shows that 372 prokaryotic hosts contain 872 caspase homologs (protein family PF00656, Peptidase C14). To obtain a deeper appreciation of the functional diversity and taxonomic distribution of caspase homologs and PCD in prokaryotes we set out to explore the 1463 finished prokaryotic genomes (downloaded from NCBI on September 23, 2011). The caspase homologs discovered were further characterized at the protein level by identification of protein domains and transmembrane helices. The data obtained revealed the existence of a wide variety of caspase homologs in prokaryotes, and their significance in the evolution of caspases and programmed cell death in pro- and eukaryotes is discussed.

## Results

### Metacaspases in the Prokaryotic Genomes

**Figure 1:**
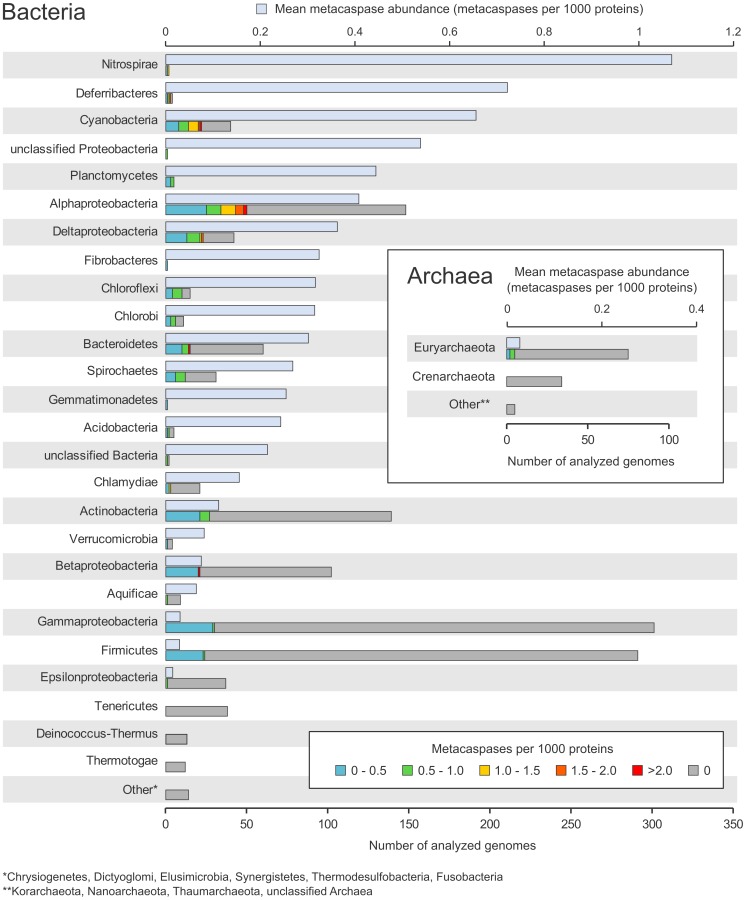
Taxonomic distribution of putative prokaryotic metacaspases in sequenced genomes. The mean metacaspase abundance (metacaspases per 1000 proteins) is shown in the upper bars (light blue), for each group (i.e. phylum, except for Proteobacteria, which have been divided into classes, unclassified Bacteria, which encompasses *Candidatus* Cloacamonas acidaminovorans and *Thermobaculum terrenum* ATCC BAA-798, and unclassified Proteobacteria, harboring the single member *Magnetococcus* sp. MC-1). The lower bars show the total number of sequenced genomes within each group. These are divided into categories based on the range of their metacaspase abundance (metacaspases per 1000 proteins). The prokaryotic groups have been ordered on mean metacaspase abundance, with the highest on top. The “Other” categories comprise groups that are represented by five or less sequenced genomes and lack putative metacaspases. Scaling is the same for both the bacterial and archaeal charts.

By searching 1463 prokaryotic genomes, using a custom bacteria-specific metacaspase HMM profile (see Methods), it was possible to identify 671 putative metacaspases. These were present in 267 strains, i.e. in 18% of the total query set. Out of these, 157 carried a single metacaspase gene, whereas 110 were equipped with two or more putative metacaspases. Five (4%) out of the 114 archaeal genomes analyzed, all belonging to the Euryarchaeota, contained one single metacaspase each. As seen in Fig. 1, the sequencing bias is pronounced among the bacterial and archaeal groups, with Gammaproteobacteria and Firmicutes holding>250 sequenced genomes. Still, a majority of the 32 bacterial groups examined include species with metacaspases, with the exception of Tenericutes, Deinococcus-Thermus, Thermotogae, Chrysiogenetes, Dictyoglomi, Elusimicrobia, Synergistetes, Thermodesulfobacteria and Fusobacteria. In addition, several of the phyla and Proteobacterial classes with few sequenced genomes harbor a high abundance of metacaspases, e.g. Nitrospirae (1.11 metacaspases per 1000 proteins), Deferribacteres (0.72) and unclassified Proteobacteria (0.54). Among the highly sequenced bacterial groups, Cyanobacteria (0.66), Alphaproteobacteria (0.41) and Deltaproteobacteria (0.36) show the highest metacaspase prevalence. In contrast, a rather low frequency of metacaspases was identified in Gammaproteobacteria (0.03 metacaspases per 1000 proteins), Firmicutes (0.03) and Epsilonproteobacteria (0.01) (Fig. 1).

A variation in metacaspase frequency within the phyla/classes examined was also obvious. Certain genomes possessed exceptionally high numbers of metacaspases. The highest was observed in *Haliscomenobacter hydrossis* DSM 1100, belonging to the phylum Bacteroidetes, with 28 genes encoding proteins with a putative metacaspase domain (corresponding to 4.15 metacaspases per 1000 proteins). Other strains with high numbers of metacaspases (≥5) are commonly found among Alphaproteobacteria, Deltaproteobacteria and Cyanobacteria (Table S1). The Alphaproteobacteria are represented by 50 strains with metacaspases, 12 of which are symbiotically competent rhizobia, while two *Sinorhizobium meliloti* strains lack metacaspase genes. Two additional Alphaproteobacteria with metacaspases have symbiotic capacity: *Methylobacterium nodulans* ORS 2060 [35] and *Methylobacterium* sp. 4–46 [36].

There is no clear correlation between developmental capacities, or confirmed PCD phenomena, and the presence of metacaspases in a particular genome. Filamentous, multicellular strains, e.g. *Catenulispora acidiphila* DSM 44928 [37], *Anabaena variabilis* ATCC 29413 [38] and *Herpetosiphon aurantiacus* DSM 785 [39] carry metacaspase genes as do some unicellular strains, e.g. *Marivirga tractuosa* DSM 4126 [40], *Thermococcus onnurineus* NA1 [41] and *Cyanothece* sp. ATCC 51142 [42]. Furthermore, strains with confirmed PCD activity may or may not possess metacaspases, e.g. *M. xanthus* DK 1622 [16], *T. erythraeum* IMS101 [14] and *X. campestris* [15], [22] that do, and e.g. *E. coli* [18] and *B. subtilis* [8] that do not.

### Domains and Domain Architectures

The catalytic cysteine-histidine (C-H) dyad of caspases is highly conserved in most taxonomic groups examined. Overall, 84% of the putative metacaspases identified in the bacterial genomes examined carry an intact C-H dyad. Furthermore, the dyad is conserved in all identified archaeal metacaspases. Six bacterial groups are distinguished by a cysteine-histidine conservation frequency lower than 90%: Gemmatimonadetes (0%, only one putative metacaspase identified in the phylum), Nitrospirae (57%), Cyanobacteria (63%), Planctomycetes (64%), Alphaproteobacteria (75%) and Spirochaetes (89%). Substitutions are equally prone to occur in the cysteine or the histidine residues. These residues are most commonly substituted by the polar serine (43% of all substitutions) and hydrophobic tyrosine (63%), respectively.

In the 671 putative prokaryotic metacaspases, 71 different Pfam domain types were identified, including the bacterial Peptidase C14 (bacterial metacaspase) domain.

**Figure 2:**
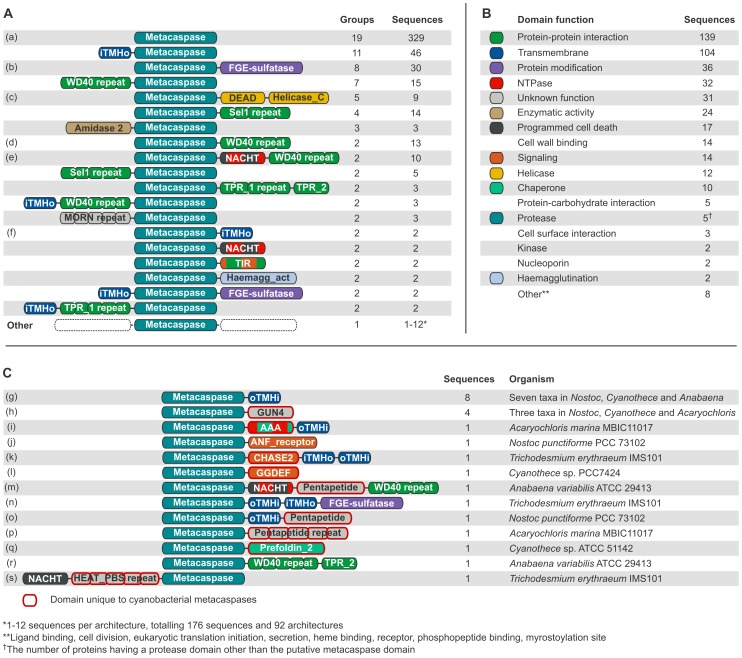
Prokaryotic metacaspase domain architectures and predicted functions. Domains and distances between domains are not drawn to scale, but the N- to C-terminal order is the same in all sequences sharing a particular domain architecture. Consecutively repeated domains have been grouped, indicated by “repeat” following the domain name. The number of repeats can differ between the sequences within one domain architecture group. “iTMHo” is a transmembrane helix with N-terminal domains predicted to be situated intracellularly and C-terminal domains extracellularly. The topology is the reverse for “oTMHi”. N-terminal transmembrane helices are likely to be signal peptides. (A) Metacaspase architectures ordered by the number of different prokaryotic groups in which they are found. The total number of sequences displaying each domain architecture is also shown. The colors reflect the function(s) of each domain and are defined in B. (B) Functional categories and color key of domains detected in addition to the metacaspase domain, ordered on the number of sequences in which they occur. Note that a single sequence, or a single domain, may have several of these functions. The function categories are based on the functional information that can be obtained from Pfam (http://pfam.sanger.ac.uk/) for each domain type. (C) Domain architectures which are unique to Cyanobacteria, and would fall into the lowermost (“Other”) category in A. Domain types which are only found in Cyanobacterial metacaspases (but may be found in other proteins in other organisms) have been emphasized by a red outline.

Out of the 398 sequences that possess only the metacaspase domain, and no other Pfam domains, 69 (17%) contain one or more transmembrane helices. The bacterial metacaspase domain was located C-terminally to a single N-terminal transmembrane helix in 46 sequences (Fig. 2A). In these cases it is possible that the predicted helix is actually a signal peptide [43]. Overall, transmembrane helices are fairly common, and are predicted to occur one or more times in 104 (15%) of the metacaspase sequences (Fig. 2B).

Taking transmembrane helices into account but disregarding consecutive repeats of domains, 112 unique domain architecture types could be identified, including single-domain proteins. 93 of the architecture variants are unique to their prokaryotic group (phylum for all but Proteobacteria, which is split into classes here), whereas 19 showed a wider distribution and may be found in two or more groups (Fig. 2A). The four Pfam domain types that occur in highest number among the putative bacterial metacaspases are WD40, TPR_1, Sel1 and TPR_2. These can be found in 10.9% (73), 7.0% (47), 6.0% (40) and 5.8% (39) of the sequences, respectively. Notably, these four domain types are all annotated as involved in protein-protein interactions (Pfam, http:/pfam.sanger.ac.uk/). FGE-sulfatase (formylglycine-generating sulfatase enzyme) is the fifth most spread domain, found in 4.9% (33) of the sequences, and catalyzes post-translational modifications of sulfatases. Polysaccharide deacetylase (2.5%, 17 sequences), NACHT (2.2%, 15 sequences) and a putative peptidoglycan binding domain (1.6%, 11 sequences) follow in abundance. The latter may be extracellular, at least in part, and would allow binding to the bacterial cell wall. Less common domain types, although occurring more than once, include ATPases, helicases, various enzymes, signaling and protein-protein interaction domains. Interesting to note are the four sequences that contain an N-acetylmuramoyl-L-alanine amidase activity (Amidase_2) domain, which belongs to a group known as peptidoglycan hydrolases. Such proteins have been implicated in autolysis, fratricide and developmental lysis processes [44]. These four sequences are found in *Polaromonas naphthalenivorans* CJ2 (Betaproteobacteria), *Nitrosococcus oceani* ATCC 19707 (Gammaproteobacteria), *Chitinophaga pinensis* DSM 2588 (Bacteroidetes) and *Desulfobacterium autotrophicum* HRM2 (Deltaproteobacteria). The *Chitinophaga pinensis* DSM 2588 sequence also carries a GDSL-like lipase (Lipase_GDSL) domain, turning this particular protein into what seems to be a highly destructive weapon, i.e. it combines lipase, amidase and protease activities. Out of the 71 domain types that are identified together with the putative bacterial metacaspase domain, 33 are particularly rare and were only found once each.

Two domain types with a link to eukaryotic PCD reside in the prokaryotic dataset: NACHT, an ATPase domain which is found in 15 sequences belonging to the groups Actinobacteria, Bacteroidetes, Chloroflexi and Cyanobacteria, and NB-ARC, a signaling motif found in two sequences from the genus *Chloroflexus* (phylum Chloroflexi). It should be noted that NACHT and NB-ARC belong to the same Pfam clan (CL0023, P-loop NTPases), as do a few other NTPase domain types identified in the putative bacterial metacaspases.

### Cyanobacterial Metacaspases

As mentioned above, a majority of the putative bacterial metacaspase architectures are unique to particular prokaryotic groups. Since Cyanobacteria provide an example of prokaryotes with developmental cycles generating phenotypes ranging from simple unicellular (genome sizes up to about 2 Mbp) to complex types with distinct cell differentiation capacities (genome sizes up to about 9 Mbp; see e.g. [45]), we investigated the 13 architecture types which are unique to Cyanobacteria (Fig. 2C). Apart from confirming the metacaspases identified earlier [34], six additional sequences were discovered (Table 1). However, some of those with low HMM coverage (<60%) may represent false positives. Looking at the full dataset of 671 putative prokaryotic metacaspases, such a low HMM coverage is only observed in 19 sequences.

**Table 1:** Cyanobacterial metacaspases identified in the present study (but not in [34]).

Organism	GI number	Domain architecture	i-E-value	HMM coverage	CH-status*
*Gloeobacter violaceus*	37519663	Bac_PepC14	1.7e-37	0.9126984127	Positive
*Trichodesmium erythraeum* IMS101	113476534	Bac_PepC14	2.90E-009	0.5515873016	Negative
*Trichodesmium erythraeum* IMS101	113475804	Bac_PepC14|FGE-sulfatase	2.90E-025	0.876984127	Negative
*Microcystis aeruginosa* NIES-843	166364442	Bac_PepC14	3.00E-011	0.2301587302	Negative
*Microcystis aeruginosa* NIES-843	166365388	Bac_PepC14	5.40E-027	0.876984127	Positive
*Microcystis aeruginosa* NIES-843	166365605	Bac_PepC14	5.40E-027	0.876984127	Positive

* “Positive” corresponds to conservation of both cysteine and histidine active site residues. “Negative” corresponds to the substitution of either or both cysteine and histidine active site residues.

Some Cyanobacterial metacaspases have domain types or architectures that are unique to Cyanobacteria (Fig. 2C). The most common unique Cyanobacterial metacaspase architecture, found in seven species, comprises a metacaspase and a C-terminal transmembrane helix, with the metacaspase domain predicted to be located intracellularly (Fig. 2C). However, the TMHMM program is able to correctly predict the precise topology of a transmembrane protein only in 77–78% of all cases, and the data should therefore be interpreted with some caution [43]. Three Cyanobacterial metacaspases incorporate GUN4 (Fig. 2C), a domain known to bind an enzyme substrate which in *Arabidopsis* affects plastid-nucleus signaling. The marine unicellular *Acaryochloris marina* MBIC11017 possesses a metacaspase domain followed by an AAA domain, which provides NTPase and chaperone activities, and a transmembrane helix (Fig. 2C). Three architectures include a domain which is implicated in signal-transduction: ANF_receptor, an extracellular ligand-binding domain, GGDEF, a domain capable of synthesizing the bacterial intracellular signaling molecule cyclic di-GMP, and CHASE2, a bacterial extracellular receptor domain (Fig. 2C). The single sequences with these architectures can be found in the unicellular nitrogen-fixing *Cyanothece* sp. PCC 7424 and the more complex, likewise nitrogen-fixing, *Nostoc punctiforme* PCC 73102 and *T.* *erythraeum* IMS101, respectively. The latter sequence is particularly interesting due to its pattern of predicted transmembrane helices (Fig. 2C). The protein consists of an N-terminal metacaspase domain and a C-terminal CHASE2 extracellular receptor domain, in turn closely flanked by four transmembrane helices, one N-terminal, and three C-terminal. The predicted topology of these transmembrane helices, together with the presence of the likely extracellular receptor domain, suggests that this is a membrane protein exposing the receptor domain to the extracellular environment and leaving the metacaspase domain free to act in the cytoplasm. Due to overlaps with the CHASE2 domain, not all predicted transmembrane helices have been added in Fig. 2C (see Methods). Other metacaspase architectures that are unique to Cyanobacteria are variations on more common architectures. First, the widely distributed Metacaspase-NACHT-WD40 (Fig. 2A) has a Cyanobacteria-specific remodelling with a Pentapeptide repeat domain (Pentapeptide) separating the NACHT domain from the WD40 repeat section (Fig. 2C). A variant of the FGE-sulfatase-containing metacaspase (Fig. 2A) is apparent, here with a pair of transmembrane helices separating the two functional domains (Fig. 2C). The Pentapetide domain, found in two more Cyanobacterial metacaspases (Fig. 2C), is specific to Cyanobacteria although of unknown function. In yet other Cyanobacteria-specific metacaspase architectures, the proteolytic domain is coupled to a chaperone, the prefoldin subunit (Prefoldin_2), or to repeated WD40 protein-protein interaction domains together with a tetratricopeptide repeat domain (TPR_2) (Fig. 2C). Finally, *T. erythraeum* IMS101 carries yet another unique Cyanobacterial metacaspase, consisting of a NACHT domain, a PBS lyase HEAT-like repeat domain (HEAT_PBS), repeated nine times, followed by the metacaspase domain.

**Figure 3:**
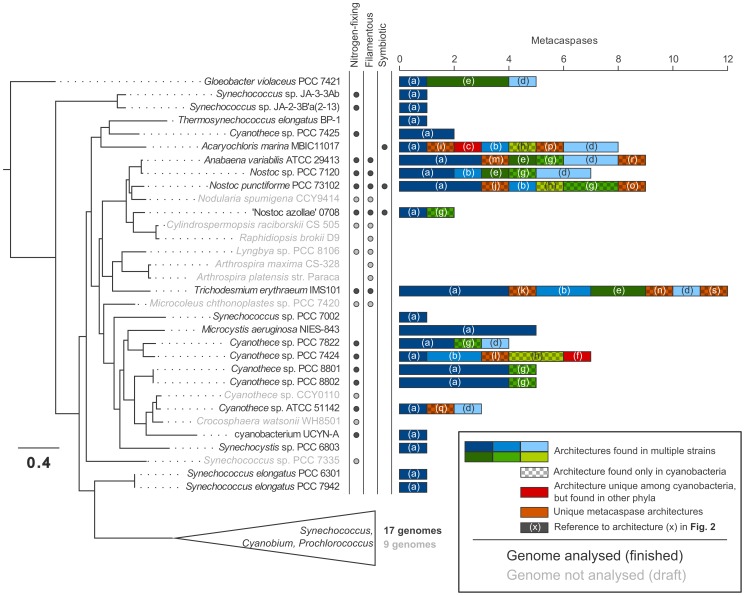
Phylogenetic distribution of Cyanobacterial metacaspases. Cyanobacterial phylogenetic tree based on 285 core orthologs, after [45]. The number of identified metacaspases have been plotted for each genome that was analyzed. The tree also shows some Cyanobacteria with draft genomes only, which were not included in this study (shaded). The clade encompassing 26 members of the unicellular genera *Synechococcus*, *Cyanobium* and *Prochlorococcus* has been collapsed as none contained metacaspases. The occurrence of the traits nitrogen fixation, filamentous morphology and symbiotic competence is shown in the figure. Letters within brackets, e.g. “(a)”, are references to the detailed domain architectures in Fig. 2.

To get a phylogenetic perspective of the Cyanobacterial metacaspases these were mapped on a phylogenetic tree based on 285 Cyanobacterial core orthologs (Fig. 3; [45]). It is apparent that metacaspases are most prevalent among filamentous nitrogen-fixing Cyanobacterial strains capable of cell differentiation, e.g. *T.* *erythraeum* IMS101 and *Anabaena*/*Nostoc*, with the exception of ‘*Nostoc azollae*’ 0708, the latter holding an eroding genome [46]. Two marine unicellular Cyanobacteria likewise show varied metacaspase/domain architectures, i.e. *Acaryochloris marina* MBIC11017 and a number of strains within the genus *Cyanothece*, while the clade comprising 26 small unicellular non-nitrogen-fixing marine strains within *Synechococcus*, *Prochlorococcus* and *Cyanobium* lack metacaspases altogether. However, the “primitive” strain at the root of the Cyanobacterial tree, *Gloeobacter violaceus* PCC 7421, is equipped with metacaspase genes (Fig. 3).

### *Xanthomonas* Metacaspases

**Figure 4:**
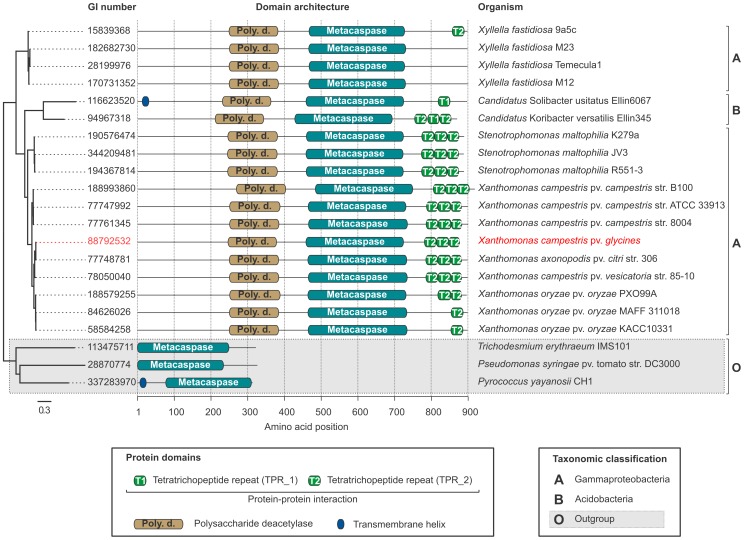
Phylogenetic relationship of polysaccharide deacetylase-containing prokaryotic metacaspases. A set of highly conserved metacaspase sequences found in the Gammaproteobacterial genera *Xyllela*, *Stenotrophomonas* and *Xanthomonas*, including *Xanthomonas campestris*, in which the protein has been implicated in programmed cell death (PCD) [15], [22], as well as in the Acidobacteria *Candidatus* Solibacter usitatus Ellin6067 and *Candidatus* Koribacter versatilis Ellin345. The metacaspase involved in *Xanthomonas campestris* pv. *glycines* PCD (GI 88792532) [68] is emphasized in red. The metacaspase domains of all 17 sequences identified in genomes analyzed in this study have conserved cysteine-histidine catalytic dyads. The maximum likelihood phylogenetic tree to the left was produced by alignment of the sequences with Muscle v3.8.31 [71] followed by tree construction with FastTree v2.1.3 [74]. Three cysteine-histidine positive sequences from *Trichodesmium erythraeum* IMS101 (Cyanobacteria), *Pseudomonas syringae* pv. tomato str. DC3000 (Gammaproteobacteria) and *Pyrocoocus yayanosii* CH1 (Euryarchaeota) were used as an outgroup.

The genus *Xanthomonas* (Gammaproteobacteria) is of special interest due to the verified correlation between caspase activity and PCD in the strain *X. campestris* pv. *glycines* AM2. Eight out of 10 analyzed *Xanthomonas* genomes harbour one metacaspase gene each, except for *X. campestris* pv. *campestris* str. B100, which has two (Table S1). The conserved domain architecture type consists of a polysaccharide deacetylase (PF01522) domain followed by the metacaspase domain and between one and three tetratrichopeptide repeat domains (PF00515, PF07719) (Fig. 4). *X. campestris* pv. *campestris* str. B100 has this architecture type (Fig. 4) and an additional metacaspase, which only has the metacaspase domain. The *Xanthomonas* metacaspases have predicted molecular weights between 97 and 100 kDa, and 35 kDa for the protein with a solitary metacaspase domain. The *Xanthomonas*-type metacaspase is also found in the Gammaproteobacterial genera *Xyllela* and *Stenotrophomonas*, as well as the Acidobacteria *Candidatus* Solibacter usitatus Ellin6067 and *Candidatus* Koribacter versatilis Ellin345 (Fig. 4). The sequences seem to be highly conserved between genera, but may have a different type of tetratrichopeptide repeat domain, or lack tetratrichopeptide repeats altogether. All 17 *Xanthomonas*-type metacaspases identified in genomes analyzed in this study have conserved cysteine-histidine catalytic dyads.

### *Haliscomenobacter* Metacaspases

*Haliscomenobacter hydrossis* DSM 1100 not only carries a large number of metacaspases (28), its domain repertoire is also highly diverse. It possesses several single-domain metacaspases, as well as more complex variants, such as the metacaspase-NACHT-WD40-repeat (Fig. 2A) and the metacaspase-FGE-sulfatase (Fig. 2A).

### Function and Localization

Multiple domains in the same protein allow assignment of function and localization to the sequences, and domain architectures may be interpreted as function combinations. The most commonly found function, beyond the metacaspase domain, is protein-protein interaction, which is present in 139 (20.7%) of the 671 putative bacterial metacaspases (Fig. 2B). Other common features are transmembrane domains (present in 104 sequences; 15.5%), protein modification (36 sequences; 5.4%), NTPase activity (32 sequences; 4.8%), various enzymatic activities (24 sequences; 3.6%), putative involvement in PCD (17 sequences; 2.5%), cell wall binding (14 sequences; 2.1%), signaling (14 sequences; 2.1%), helicase activity (12 sequences; 1.8%), chaperone function (10 sequences; 1.5%), protein-carbohydrate interaction (5 sequences; 0.7%) and proteolytic activity (5 sequences; 0.7%). A domain with an unknown function was found in 31 sequences and some domains have multiple functions, as defined here. For instance, some helicases may also have NTPase activity, and signaling is in some instances coupled to protein-protein interaction (e.g. TIR; Fig. 2A).

Cellular localization could be established, as being intracellular, membrane-associated or extracellular, only in a fraction of the metacaspase-linked domain types. Not less than 529 out of 671 sequences completely lacked this kind of information. The remainder may be grouped into four categories: Membrane (98 sequences), of which the majority possess an N-terminal transmembrane helix that might in fact be a signal peptide, Extracellular (30 sequences), Intracellular (7 sequences) and Membrane and extracellular (7 sequences).

### Metacaspases in Operons

By employing the MicrobesOnline operon database (http://www.microbesonline.org/operons/, [47], [48]), with data on predicted operons in 200 out of the 267 genomes found to harbor metacaspases, we found that 128 of these had at least one metacaspase in a predicted operon. Out of the grand total of 508 identified metacaspases in the 200 genomes, 237 (46.6%) were distributed over 217 operons. More than one metacaspase, up to a maximum of three (in *Desulfococcus oleovorans* Hxd3), were found in 20 operons. Only four bacterial groups had such multi-metacaspase operons, namely Alphaproteobacteria, Bacteroidetes, Cyanobacteria and Deltaproteobacteria.

Operon neighbors were analyzed using the same HMM-based Pfam domain identification approach as was used for the metacaspases themselves (see methods). Together with the original annotation of the neighboring sequences, this allowed the addition of another layer of functional annotation to the metacaspases. Consequently, a highly diverse assortment of functions where attributed to the operons. Most conspicuously, various enzymes were found in 35 operons. The most common types were different hydrolysases, an enzyme involved in antibiotic resistance (Lactamase B), and a sulfate metabolism enzyme (ATP-sulfurylase) specific to Cyanobacteria in this dataset. Less common enzymes were linked to e.g. carbon and lipoprotein metabolism. Protein-protein interaction domains and signaling, sensor and regulatory domains were found in 30 and 23 operons, respectively, while domains implicated in proteolysis (not counting any bacterial Peptidase C14 domains) were found in 23 operons. DNA interaction, as performed by for example DNA binding domains, various nucleases and a recombination domain, was identified in 18 operons. Three of these operons appeared to also be connected to restriction activity. Sixteen metacaspase operons are involved in functions at the cell surface, as indicated by e.g. transporters, flagellar proteins, surface antigens and cell wall binding domains. OmpA, an outer membrane-associated protein domain whose precise function is unclear, occurs in 14 operons.The core processes transcription and translation were linked to 15 and 11 operons, respectively. Domain examples include RNA polymerase subunits, sigma factors, tRNA processing proteins and even ribosomal proteins. Less common functions that were inferred from the domains were e.g. protein modification, chaperone activity and secretion. Another rare occurrence was the finding of the PCD-connected NACHT domain in a non-metacaspase protein in the operon identified in *Roseiflexus castenholzii* DSM 13941 (Chloroflexi). Furthermore, an N-acetylmuramoyl-L-alanine amidase domain, which can play a role in bacterial autolysis [44], [49], was found in the operon of *Geobacter sulfurreducens* PCA (Deltaproteobacteria). Despite all this data, the function remains unknown for 70 out of the total of 217 metacaspase-containing operons.

**Figure 5:**
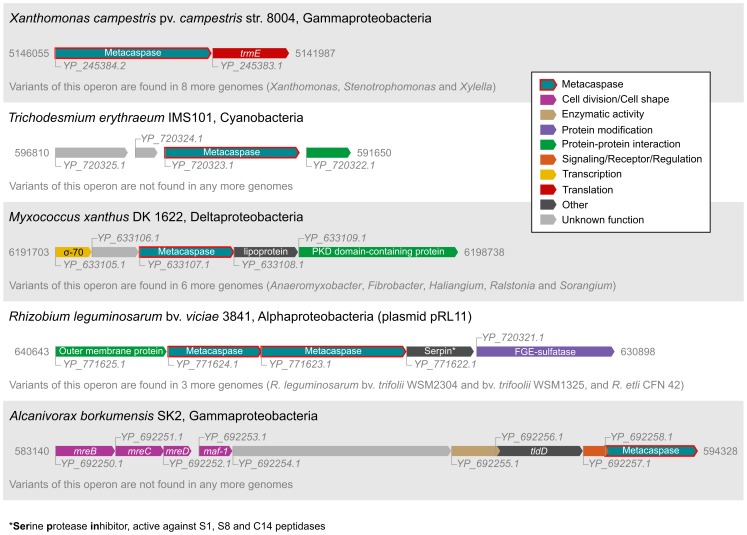
Examples of operons with metacaspase genes. Operon structures were obtained from the MicrobesOnline operon database (http://www.microbesonline.org/operons/, [47], [48]), supplemented with genomic region data from NCBI (http://www.ncbi.nlm.nih.gov/gene). The numbers to the left and right of each operon are the start and end nucleotide positions. The start is higher than the end when the operon is situated on the minus strand. GenBank accession IDs are shown at the beginning of each gene. The protein products of the *Trichodesmium erythraeum* IMS101 and *Xanthomonas campestris* pv. *campestris* str. 8004 metacaspase genes are displayed in Fig. 2C and Fig. 4, respectively.

Certain operon configurations appeared to be conserved between strains, e.g. an operon variant with protease and protease inhibition activities found in four *Rhizobium* strains (Fig. 5). Another eight *Clostridium botulinum* metacaspase operons appeared to be phage-related, and possibly of similar origin, although their lengths varied between 11 and 18 genes. The *Xanthomonas*-type metacaspase operon also seems to be conserved, as the homologous metacaspases in *Xanthomonas*, *Xylella* and *Stenotrophomonas* have similar operon structures with an apparent function in translation, i.e. the neighboring tRNA modification GTPase TrmE gene. While the operon consists of only two genes in *Xanthomonas* and *Stenotrophomonas* (Fig. 5), *Xyllela* adds two upstream genes: ribonuclease P (*rnpA*) and inner membrane protein translocase component *yidC*. These genes are found in the same configuration also in the other two genera, but were not predicted to be in the same operon. Another seemingly conserved configuration is found in the four *Myxococcus xanthus* DK 1622 metacaspase operons, which thereby includes four of the total of five metacaspases in this genome. All of these operons share a common denominator, i.e. sigma factors (sigma-70), placing them in the DNA interaction and transcription categories. This operon structure also appears in genomes of the other Deltaproteobacterial genera *Anaeromyxobacter*, *Haliangium* and *Sorangium*, but also in *Ralstonia* (Betaproteobacteria) and *Fibrobacter* (Fibrobacteres).

Lastly, a particularly interesting operon, in the light of discussing metacaspases in connection to PCD and development, is the one found in *Alcanivorax borkumensis* SK2 (Fig. 5). In this, a metacaspase gene is found together with homologs of the genes *mreBCD* and *maf-1*, which are involved in cell division and cell shape determination [50]–[52].

Raw data on all identified putative prokaryotic metacaspases, and their operons when applicable, is available as supplementary information (Dataset S1 and Table S2).

## Discussion

It is apparent from our analyses that metacaspase-encoding genes show a wide taxonomic distribution and that they appear in a majority of bacterial phyla examined as well as among archaea (Euryarchaeota), although at a considerably lower frequency in the latter. Nevertheless, it is also clear that a minority of the sequenced prokaryotes, about one fifth, carry genes encoding these proteases. However, there is a significant phylogenetic distribution bias among sequenced genomes, as also highlighted earlier [53]. Sequencing attempts have primarily focused on cultured microbes isolated from specific habitats and with special physiological traits, or those related to previously sequenced bacteria. Future sequencing efforts should preferentially be phylogeny-driven [53] and include global metagenome survey data, providing access to the vast majority of unculturable microbes [54]–[57]. Using a bacterial phylogenetic tree based on an alignment of 31 conserved protein-coding genes [53] as a background, it became obvious that sequenced genomes of the early-branching groups, Deinococcus-Thermus, Thermotogae and Synergistetes, are all devoid of metacaspase genes. However, the low number of available genomes could obscure the true abundance of metacaspases within these phyla. The same apparent lack of metacaspases was observed in Fusobacteria and Tenericutes. Notably, these constitute the sister group [53] of Firmicutes, Chloroflexi, Cyanobacteria and Actinobacteria, which all include species with metacaspases. The six latter phyla in turn form a clade which is the sister group of all remaining bacterial phyla, all with species carrying metacaspase genes. The scattered metacaspase gene distribution within the prokaryotic sphere may be the result of gene losses or perhaps more likely of gene gains (e.g. via horizontal gene transfer among prokaryotes), the latter followed by metacaspase diversifications during the continued evolution of the prokaryotes. Extensive horizontal gene transfer could also explain the presence of metacaspases with similar architectures or operon structures in separate bacterial groups (Fig. 2A and Fig. 5). Most probably, however, no single evolutionary process will be solely accountable, and differential retention and deletion of DNA originating from ancient duplication and paralog evolution events could play a role as well. Further analyses, e.g phylogenetic, would allow elucidation of the evolutionary mechanisms that have shaped metacaspases and their dispersion in prokaryotes, preferably aided by less biased genomic databases.

The comparatively low incidence of metacaspase genes (18% of genomes) discovered among the sequenced prokaryotes combined with their heterogeneous taxonomic distribution suggest that only certain prokaryotic life strategies require, or benefit from, the employment of metacaspases and/or PCD. Further, the fact that for instance *B. subtilis* [8] and *E. coli* [18] perform PCD in the absence of metacaspase genes underpins the hypothesis that metacaspases are not prerequisites for prokaryotic PCD. Caspase type proteases, including those in eukaryotes, may in general therefore have originated from within less basal bacteria or archaea, equipped with wide-spread and highly diverse types of metacaspases. Indeed, the eukaryotic metacaspases are thought to have been introduced via the prokaryotic ancestor that, through a monophyletic endosymbiotic event, gave rise to mitochondria [33], based on phylogenetic studies of a limited number of caspase homologs. The origin of paracaspases and caspases is less clear, but a horizontal gene transfer from Alphaproteobacteria has been proposed [33].

A few of the more extensively sequenced bacterial groups, namely Alphaproteobacteria, Deltaproteobacteria and Cyanobacteria, display pronounced metacaspase abundance. The metacaspase-endowed Alphaproteobacteria are to a great extent rhizobial species, e.g. *Bradyrhizobium japonicum* USDA 110 and *Rhizobium leguminosarum* bv. *trifolii* WSM1325, that enter nitrogen-fixing symbioses with legumes [58], [59]. Two other Alphaproteobacterial strains with a large number of metacaspases, *Methylobacterium nodulans* ORS 2060 and *Methylobacterium* sp. 4–46, are likewise symbiotically competent [35], [36], while non-competent strains have a lower number (Table S1). Add to this that the mitochondrial ancestor is assumed to have carried metacaspases (see above) as do extant symbiotically competent Cyanobacteria (*Acaryochloris marina* MBIC11017, *Nostoc punctiforme* PCC73102 and ‘*Nostoc azollae*’ 0708), and potentially also the ancestor of plant chloroplasts [46], [60], [61]. It is therefore tempting to speculate that metacaspases may have a role in the evolution of eukaryotes and in the establishment or the maintenance of a symbiotic life style, but as not all rhizobia carry caspase homologs (two *Sinorhizobium meliloti* strains are lacking) the latter may reflect a mere coincidence or the sequencing bias. It is however known that the role of metacaspases may go beyond that of PCD processes [25].

PCD processes play a crucial role in for instance mammalian and plant development [3], [62]. The Deltaproteobacteria harbour *Myxococcus* species that possess metacaspase genes, i.e. *M. fulvus* HW-1 and *M. xanthus* DK 1622 (Table S1), and *M. xanthus* is known to go through a tightly regulated cell death program, involving a protein kinase cascade and the MazF-mx ribonuclease. Downstream effects lead to the destruction of 80% of the population, concurrent with the development of fruiting bodies and myxospores [16], [21]. It has been suggested that this could involve autolytic enzymes [16] and it is conceivable that the metacaspases identified here could represent such a group of enzymes. The implications of this finding might even stretch outside of the genus *Myxococcus*, as a conserved metacaspase operon was also present in other bacteria (Fig. 5).

Another metacaspase-possessing organism of the Deltaproteobacteria is *Alcanivorax borkumensis* SK2, which degrades oil and forms biofilms to facilitate the former [63], [64]. As biofilm formation may involve PCD [12], [19], it is interesting to note that the metacaspase gene of this bacterium is found in an operon including also the cell shape and cell division genes *mreBCD* and *maf-1* (Fig. 5)[50]–[52], indicating that this operon might be involved in developmental processes, which could also be true for the metacaspase.

The distribution of metacaspases among Cyanobacteria follows a clear pattern, that may also relate metacaspases (and PCD) to genotypic and/or phenotypic complexity. All species that lack metacaspases belong to a monophyletic clade of extremely wide-spread marine planktonic and unicellular Cyanobacteria with small genomes (Fig. 3), i.e. *Synecchococcus* and *Prochlorococcus*, whereas metacaspases are highly abundant and show varied domain architectures in the clade composed of Cyanobacteria with larger genomes exhibiting more complex morphologies and/or physiological capacities. For instance, yet another globally wide-spread marine planktonic Cyanobacterium, *T. erythraeum* IMS101, possesses the richest metacaspase abundance and domain architectures among Cyanobacteria. This verifies the evolution of totally different life style preferences within the Cyanobacterial radiation even among Cyanobacteria sharing the same environment, as also suggested earlier [45]. Overall, metacaspase numbers and variants are pronounced among the Cyanobacteria capable of cell differentiation (*Nostoc punctiforme* PCC 73102, *Anabaena variabilis* ATCC 29413 and *Nostoc* sp. PCC 7120) which suggests a link between developmental complexity and the presence of metacaspases. Exceptions are the plant endosymbiont ‘*Nostoc azollae*’ 0708, however this strain holds a proven eroding genome [46], as are two marine unicellular genera *Cyanothece* and *Acaryochloris*, however both possess comparatively larger genomes reflected in a wider repertoire of capacities (e.g. nitrogen fixation and the symbiotic competence, respectively) compared to other unicellular representatives [65], [66]. Interestingly, the phylogenetic distribution of metacaspases within the Cyanobacterial radiation suggests that the ancestor of the Cyanobacteria carried at least one metacaspase gene, as do still *Gloeobacter violaceus* PCC 7421, a ‘primitive’ Cyanobacterium with rudimentary thylakoids (Fig. 3). This suggests that metacaspases originated early in the evolution of life (∼3 B.y.a.). Metacaspases would then later have been lost among members of the *Synechococcus* and *Prochlorococcus* clade, in line with their extensive genomic streamlining and life style [45], [67], while some other relatives, such as hot spring *Synechococcus* and *Synechocystis*, still possess one metacaspase each.

Although metacaspases are generally quite scarce among Gammaproteobacteria (Fig. 1), the genus *Xanthomonas* provides a remarkable exception showing correlation between PCD and caspase expression and activity [15], [22]. A combined polysaccharide deacetylase and caspase gene (GI 88792532) exists in *X. campestris* pv. *glycines*, whose product cross-reacted with both caspase and PARP (poly ADP-ribose polymerase) antibodies [68]. The polysaccharide deacetylase domain even displayed homology to human PARP-1, whose cleavage by caspase-3 is a characteristic of apoptosis. Furthermore, the *X. campestris* pv. *glycines* gene (GI 88792532) is homologous to genes identified in this study (Fig. 4), which suggests they could also be connected to PCD in bacteria. The size of the identified caspase enzyme was 55 kDa [22], whereas the ones of the polysaccharide deacetylase-containing type identified in the present study (Fig. 4) were about 100 kDa. This discrepancy could be due to post-translational processing in the form of proteolytic cleavage, perhaps through auto-processing, which would also fit with the observation of an apparent lag phase between caspase expression and activation of the enzymatic activity [22]. The presence of these *Xanthomonas*-type metacaspases and the accompanying conserved operon (Fig. 5) in several bacterial genera, including *Xanthomonas*, *Stenotrophomonas* and *Xyllella*, warrants further investigation at the functional level to determine their physiological role.

Finally, the sheer number of metacaspases (28) encoded by the genome of *Haliscomenobacter hydrossis* DSM 1100 is striking. This organism, which grows in wastewater treatment plants [69], has a distinct filamentous morphology, with cells predominantly occurring in chains and individual cells sometimes exhibiting a branching phenotype [70]. However, if and to what extent these metacaspases are related to PCD is not known.

We further discovered a strikingly high number of metacaspases with the conserved catalytic amino acid dyad substituted by other amino acid residues in both Cyanobacteria and Alphaproteobacteria. This could indicate that the metacaspases of these groups have been subject to a longer and more eventful evolutionary history, giving rise to a multitude of paralogs, some of which may be adapted to perform functions that do not require an intact catalytic site. For instance, it is known from mammals that caspase homologs lacking catalytic residues may even function as negative caspase activity regulators [24]. Add to this that the prokaryotic metacaspases identified here seem to have become adapted to perform a wide range of functions, judging from their varied domain architectures, which suggest roles as disparate as enzymatic activity, signaling, chaperone activity and helicase function (Fig. 2). This impression is retained when taking the operon structures into account, as the neighboring genes are engaged in a variety of activities, such as hydrolysis, carbon metabolism, signaling, transport across membranes, and even in fundamental cellular processes such as transcription and translation. Moreover, as metacaspase domains were found joined to formyl-glycine generating sulfatase enzyme (FGE-sulfatase) in 33 sequences (see Results and Fig. 2), this combination seems to play an important role, and it may be speculated that both a proteolytic and a formyl-glycine modification is required to facilitate some still unknown process. Yet another four sequences were found to combine metacaspase and N-acetylmuramoyl-L-alanine amidase activity (Amidase_2) (Fig. 2A). These peptidoglycan hydrolases cleave critical constituents of bacterial cell walls, and amidases have been shown to act in antibiotic-induced cell death in *Streptococcus aureus* [49]. Furthermore, the implication of peptidoglycan hydrolases in autolysis, fratricide and developmental cell death, e.g. in *Myxococcus xanthus* and *Bacillus subtilis* [44], strengthens the case for a PCD role of these bacterial metacaspases. The presence of numerous, small protein-protein interaction domains in many prokaryotic metacaspases could also be indicative of a connection to PCD, as such domains are known important elements in metazoan apoptosis pathways [27]. Apoptotic ATPases are commonly found in proteins separate from caspases [33]. Here, the NACHT domain was identified in numerous bacterial metacaspases (Fig. 2A), and other NTPase domains, which could perform similar molecular functions, were also common. Together with the detection of two NB-ARC-containing metacaspases, these findings stress the involvement of at least some prokaryotic metacaspases in PCD.

In addition, many metacaspase-associated domains seem to have an extracellular localization, as also indicated by the presence of N-terminal transmembrane helices (Fig. 2). Extracellular localization could mean that the protein would perform its role in PCD on the surface of the cell, or engage neighboring cells in a fratricidal fashion.

### Conclusions and Future Prospects

We have demonstrated a limited (18%) but wide-spread taxonomic distribution of prokaryotic caspase homologs (metacaspases) among the prokaryotes examined, including archaea, and that these seem to play a variety of roles, both PCD- and non-PCD-related, based on the abundant protein domain architectures and operon structures discovered. In Cyanobacteria, a correlation was apparent between the presence of metacaspases and complex prokaryotic life styles, and *T. erythraeum* IMS101 has also been shown to undergo PCD [14]. In contrast, metacaspases were absent in Cyanobacterial representatives basing their life style on streamlined small genomes.

To further our knowledge on the occurrence and role of prokaryotic caspase homologs, including and beyond PCD, some intriguing avenues are worth exploring. These include to what extent the many metacaspase genes found may have any links to symbiotic events, the role of the *Xanthomonas*-type metacaspase outside of the genus *Xanthomonas,* and even outside the class Gammaproteobacteria, the function of the metacaspase in the cell division/cell shape operon of *A. borkumensis* SK2 and the significance of the stunning 28 metacaspase genes in *H. hydrossis* DSM 1100. Yet another interesting aspect to explore is the evolution of metacaspases within bacteria and archaea and especially their relation to the evolution of eukaryotes and the use of PCD in e.g. structuring body plans of metazoans. To achieve this it is now essential to include the metagenomes of the vast and untapped natural microbiome populations, such as those from terrestrial and aquatic habitats and not least the human microbiome.

## Materials and Methods

### Construction of a Bacteria-specific Metacaspase Hidden Markov Model Search Profile

Bacterial protein sequences belonging to the Pfam-A 25.0 (http://pfam.sanger.ac.uk/) protein family Peptidase_C14 (PF00656) were used to train a bacteria-specific metacaspase Hidden Markov Model (HMM) search profile. The Pfam Peptidase_C14 profile HMM and the HMMER 3.0 (http://hmmer.janelia.org/) program hmmsearch were used to identify the Peptidase_C14 domains in the selected protein sequences. The Peptidase_C14 domains, defined by the HMMER domain envelope, were then aligned using MUSCLE v3.8.31 [71]. The alignment was manually inspected and edited by removing positions of low quality at the start and end, and ensuring that the cysteine and histidine catalytic dyad residues were properly aligned. Sequences lacking one or both of the catalytic residues were discarded. The aligned Peptidase_C14 domains were clustered at 90% identity using the software cd-hit [72], [73]. The longest sequence in each cluster was then selected to form a group of cluster representatives, in order to reduce bias due to the presence of multiple highly similar sequences in the data set. The selected domains were aligned again, followed by manual inspection and editing, using the same approach as previously described. The alignment was then used to produce the bacteria-specific metacaspase HMM search profile, by applying the program hmmbuild, from the HMMER 3.0 suite, at default settings.

### Improved Search Profile

Comparison of the E-value distributions and number of significant hits when searching prokaryotic genomes indicated that building a bacteria-specific search profile was an improvement from the original Pfam Peptidase_C14 search profile, for prokaryotic genomes. That is, it was able to produce hits with lower E-values overall (Fig. S1), and added more than 50 putative metacaspases to the results. This came at the expense of losing four hits that could only be identified by Pfam’s original Peptidase_C14 profile HMM.

### Identifying Metacaspases in Sequenced Prokaryotic Genomes

FASTA files representing 1463 finished prokaryotic genomes were downloaded from NCBI (ftp://ftp.ncbi.nih.gov/genomes/) on September 23^rd^ 2011. Metacaspases in these genomes were identified using the bacteria-specific metacaspase profile HMM and HMMER 3.0 hmmsearch at default settings. Sequences with a domain independent E-value ≤0.01 and a score/bias ratio ≥10 were accepted.

### Analyzing the Domain Architectures of Identified Metacaspases

The identified, full-length metacaspase sequences were subjected to a HMMER 3.0 hmmscan search at default settings, using the Pfam 25.0 protein family database of profile HMMs, supplemented with the bacteria-specific metacaspase profile HMM produced earlier. In addition, the sequences were analyzed for the presence of transmembrane helices using TMHMM v2.0 [43]. The metacaspase domains, identified using the bacteria-specific metacaspase profile HMM and defined by the HMMER domain envelope, were extracted and aligned using MUSCLE v3.8.31, followed by manual inspection, in order to assess the conservation status of the catalytic cysteine-histidine dyad. The domain architecture, i.e. the order of domains along the N- to C-terminal axis, of each metacaspase candidate was determined using the output data from hmmscan and TMHMM v2.0. Only domains with a domain independent E-value ≤0.01 and a score/bias ratio ≥10 were accepted. If a transmembrane helix would occupy the same region as a Pfam domain, the Pfam domain would take precedence. Whenever two Pfam domains, defined by the domain envelopes determined by HMMER, overlapped more than 25% of the length of either one of them, only the domain with the lowest E-value was accepted. Thus, a comprehensive record of the domain architectures of the prokaryotic metacaspases could be created. A sequence was accepted as a metacaspase only if its final architecture contained the bacterial metacaspase domain, after contending with other domain types identified in the same region.

The domain architecture of the *Xanthomonas campestris* pv. *glycines* metacaspase (GI 88792532) was analyzed in the same manner as the metacaspases identified in the genomes, but excluding alignment of the metacaspase domain for investigation of the cysteine-histidine dyad conservation.

### Functional Classification

In order to get a better grip on the functions of the identified domains, and thereby the functions of the metacaspase proteins, the information available on the Pfam website (http://pfam.sanger.ac.uk/) was consulted, which includes additional information from InterPro (http://www.ebi.ac.uk/interpro/). The Pfam release used for the functional annotation was 26.0. Each domain type found in the metacaspase data set was looked up and assigned a specific function category and cellular localization, if possible. In some cases it was not possible to find a clearly defined function, or no function had been assigned at all. These domains were determined to have an “Unknown function”.

### Identifying Metacaspase Operons

Data on predicted operons in metacaspase-containing genomes covered by the MicrobesOnline operon database (http://www.microbesonline.org/operons/, [47], [48]), i.e. 200 out of the 267 genomes with metacaspases, was downloaded on August 28^th^ 2012. Operons containing metacaspases were identified, and FASTA files for the neighboring sequences were downloaded from NCBI. Domain architectures for these sequences were obtained using the same method as for the metacaspases (see above). Functional classification of each operon was carried out by manual inspection, aided by gene names and information about identified Pfam domains (see “Functional classification” above).

### Calculation of Molecular Weight

The molecular weight of proteins carrying a polysaccharide deacetylase domain, such as those found in *Xanthomonas campestris*, was calculated using the ExPASy “Compute pI/Mw tool” (http://web.expasy.org/compute_pi/).

### Taxonomic Distribution

Taxonomic classifications from NCBI (http://www.ncbi.nlm.nih.gov/taxonomy) were used to determine the distribution of the identified putative metacaspases among the 1463 genomes. Proteobacteria were divided into classes to provide better taxonomic resolution.

## Supporting Information

Figure S1 **Comparison of the E-values of hits yielded by two caspase-trained Hidden Markov Model search profiles.** The distribution of the log10-transformed domain independent E-values of hits in prokaryotic genomes identified when searching with the custom bacterial metacaspase profile Hidden Markov Model (HMM) and the original Pfam caspase (PF00656) profile HMM.
(TIFF)

Table S1 Metacaspase distribution among analyzed genomes.
(XLS)

Table S2 Metacaspases in operons.
(XLS)

Dataset S1 **Details of the identified prokaryotic metacaspases.**
(TXT)
