# Supplementary figures and images for: Prokaryotic Caspase Homologs: Phylogenetic Patterns and Functional Characteristics Reveal Considerable Diversity
Source: PLoS One. 2012 Nov 19;7(11):e49888. doi: 10.1371/journal.pone.0049888 (PMC3501461; doi:10.1371/journal.pone.0049888)

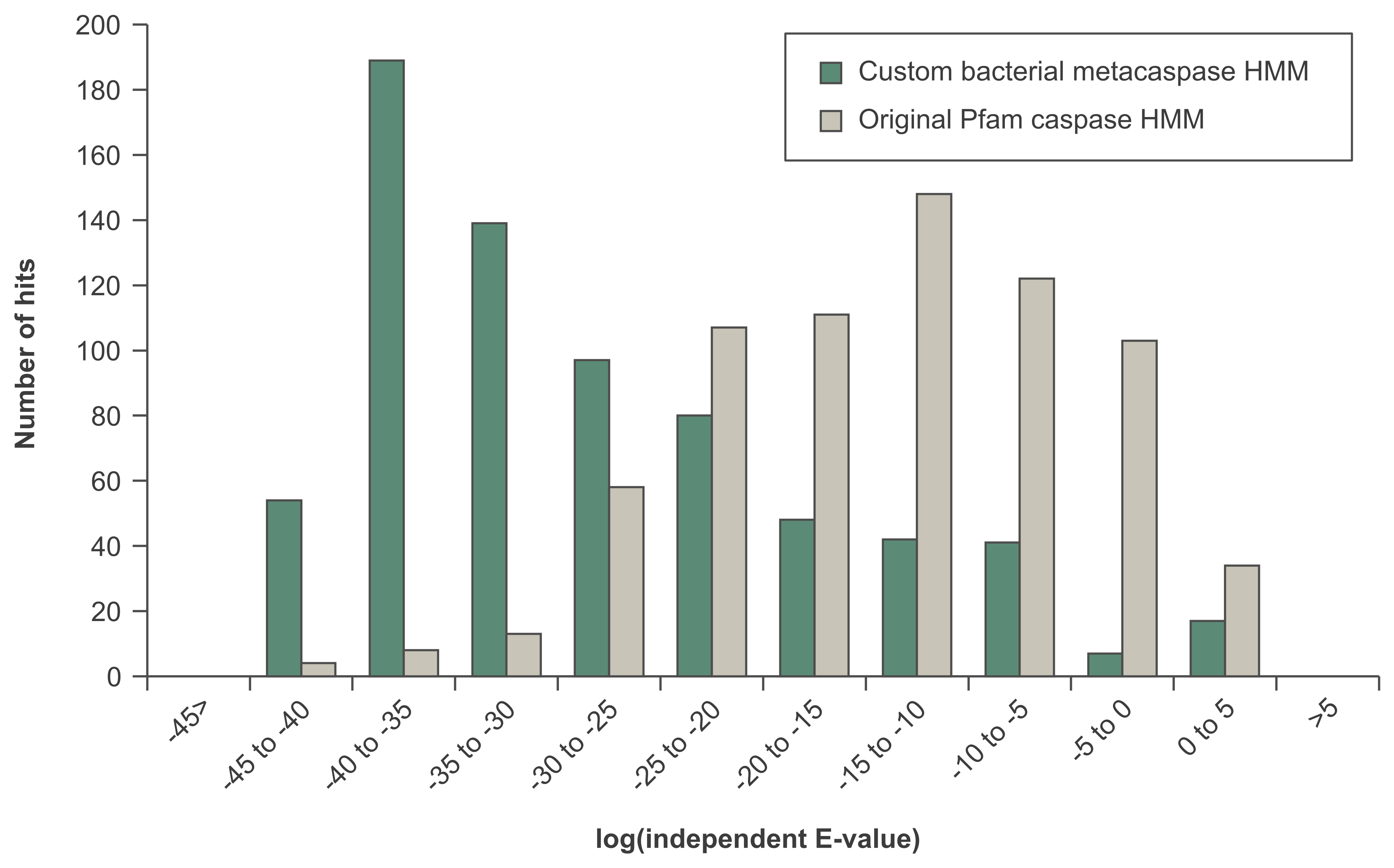

Supplement: Figure S1 — Comparison of the E-values of hits yielded by two caspase-trained Hidden Markov Model search profiles. The distribution of the log10-transformed domain independent E-values of hits in prokaryotic genomes identified when searching with the custom bacterial metacaspase profile Hidden Markov Model (HMM) and the original Pfam caspase (PF00656) profile HMM. (TIFF) [file pone.0049888.s001.tiff]
